# Safety and efficacy of an oxycodone vaccine: Addressing some of the unique considerations posed by opioid abuse

**DOI:** 10.1371/journal.pone.0184876

**Published:** 2017-12-01

**Authors:** M. D. Raleigh, S. J. Peterson, M. Laudenbach, F. Baruffaldi, F. I. Carroll, S. D. Comer, H. A. Navarro, T. L. Langston, S. P. Runyon, S. Winston, M. Pravetoni, P. R. Pentel

**Affiliations:** 1 Minneapolis Medical Research Foundation, Minneapolis, MN, United States of America; 2 Research Triangle Institute, Research Triangle Park, NC, United States of America; 3 New York State Psychiatric Institute and Department of Psychiatry, College of Physicians and Surgeons of Columbia University, New York, NY, United States of America; 4 Winston Biopharmaceutical Consulting, Boulder, CO, United States of America; 5 Department of Medicine, University of Minnesota Medical School, Minneapolis, MN, United States of America; 6 Center for Immunology, University of Minnesota Medical School, Minneapolis, MN, United States of America; Scripps Research Institute, UNITED STATES

## Abstract

Among vaccines aimed at treating substance use disorders, those targeting opioids present several unique medication development challenges. 1) Opioid overdose is a common complication of abuse, so it is desirable for an opioid vaccine to block the toxic as well as the addictive effects of opioids. 2) It is important that an opioid vaccine not interfere with the action of opioid antagonists used to reverse opioid overdose or treat addiction. 3) Some opioids are immunosuppressive and chronic ongoing opioid use could interfere with vaccine immunogenicity. 4) Although antibody-bound oxycodone is unable to enter the brain because of its size, it might still be able to activate peripheral opioid receptors. To assess vaccine impact on opioid toxicity, rats vaccinated with oxycodone conjugated to keyhole limpet hemocyanin subunit dimer (OXY-dKLH) adsorbed to alum or controls vaccinated with dKLH were compared with regard to oxycodone-induced hotplate analgesia and oxycodone-induced respiratory depression and bradycardia. Vaccination shifted the dose-response curves to the right, representing protection, for each of these endpoints. Naloxone was equally effective in both OXY-dKLH and control groups, providing complete and rapid reversal of respiratory depression. The administration of a long-acting naltrexone formulation during vaccination did not impair vaccine immunogenicity in mice. Similarly, serum anti-oxycodone antibody titers were not altered by continuous morphine infusion during vaccination compared to opioid-naïve controls. Competitive ELISA assay showed negligible or low affinity of immune antiserum for endogenous opioids or opioid antagonists. In vitro receptor binding assays showed that antibody-bound oxycodone does not activate mu opioid receptors. These data support further study of OXY-dKLH as a potential treatment for oxycodone abuse and suggest that vaccination might also reduce the severity of oxycodone overdose.

## Introduction

Vaccines have been studied as potential adjuncts for the treatment of substance use disorders including nicotine [1], cocaine [2], methamphetamine [3] and opioids [4]. Key to their efficacy is their ability to elicit high concentrations of high affinity antibodies against those drugs [5, 6]. Vaccines targeting opioids present some unique considerations with regard to their safety or efficacy. Above and beyond the enormous personal and societal harm of opioid addiction, opioid abuse is now a leading cause of accidental death in the U.S [7, 8]. This presents a complex clinical setting for the possible use of a vaccine as a treatment strategy.

Opioid vaccines have been developed with the goal of blocking the addictive effects of opioids, most importantly their reinforcing and rewarding properties [4]. Less attention has been directed at blocking their toxic effects such as respiratory depression, bradycardia, or coma. In principle a vaccine should block all opioid effects since it acts by binding the targeted opioid and preventing its distribution to the central nervous system as well as its interaction with opioid receptors. However toxic effects of opioids tend to occur at higher doses than reinforcement or reward, and may to some extent be mediated outside of the central nervous system. It is therefore important to establish whether opioid vaccines provide protection against opioid toxicity, most importantly respiratory depression, as well as addiction. If this were not the case, vaccinated individuals using large doses of oxycodone to get the desired reward might experience disproportionate respiratory depression.

While the goal of an opioid vaccine is to block opioid agonist effects, it is important to preserve the efficacy of opioid antagonists. Naloxone is widely used both by medical personnel and bystanders to reverse opioid overdose [9] while naltrexone is an effective treatment option for opioid use disorder [10]. Because these uses of opioid antagonist are critical it is important that an opioid vaccine not interfere with their clinical use.

Opioids are well recognized to interact and in some cases interfere with immune responses. Morphine, the best studied in this regard, has been shown to blunt antibody responses to several infectious agents or immunogens in animals [11]. Opioid addicts or those on methadone maintenance may show mildly reduced titers to hepatitis B and influenza vaccines [12, 13]. It is unclear to what extent impaired vaccine responses might be due to opioid use *per se* or to other conditions common in opioid abusers such as infections or the use of other drugs. Nevertheless it is possible that ongoing opioid use could blunt the generation of antibodies from an opioid addiction vaccine. If so, this could limit the efficacy of an opioid vaccine or require a more aggressive vaccination regimen with higher immunogen doses or more booster doses.

Addictive drugs are small molecules and it is assumed that once they bind to antibody they are sterically hindered from interacting with drug receptors, but this assumption has not been tested explicitly. Opioids (MW 300–500 Da) are somewhat larger than nicotine, cocaine or amphetamines (MW 149–303 Da), so it is reasonable to ask whether opioid binding to antibody prevents them from activating opioid receptors. In animals, vaccination with heroin or oxycodone vaccines blocks or attenuates a variety of behaviors that are mediated primarily by mu opioid receptors (MOR) [14–17]. This argues that binding to antibody prevents opioid binding to the MOR. However it is difficult to distinguish from these data alone whether complete or partial inhibition of opioid binding to the MOR has occurred. Serum opioid concentrations in vaccinated subjects can be quite high because of binding of opioid to the very high levels of opioid-specific antibody present in serum [18]. It is therefore of interest to assess the extent to which opioid binding to antibody prevents activation of the MOR.

The current studies in rats and mice addresses these concerns using a well characterized oxycodone vaccine that has been shown to reduce oxycodone distribution to brain in rodents and attenuate addiction-relevant behaviors including oxycodone-induced antinociception and self-administration [17–19]. The issues addressed in this study are among the most important challenges posed by the development of opioid vaccines.

## Materials and methods

### Ethics statement

This study was carried out in strict accordance with the recommendations in the Guide for the Care and Use of Laboratory Animals of the National Institutes of Health. Animal protocols were approved by the Minneapolis Medical Research Foundation Animal Care and Use Committee. All surgery was performed under isoflurane anesthesia, animals were euthanized by CO_2_ inhalation using AAALAC approved chambers, and all efforts were made to minimize suffering.

### Animals

Male Holtzman rats (Envigo, Madison, WI) weighing 200–225 g were double housed with 12/12-hour standard light/dark cycle. Male BALB/c mice (Envigo, Madison, WI) 5–6 weeks old were housed in groups of 4 under 12/12-hour standard light/dark cycle. Rats were used for most studies because oxycodone vaccine effects have been most extensively studied in this species. Mice were used for the morphine immunosuppression experiment because this model is well established in mice [20]. Mice were used for the naltrexone immunosuppression experiment because naltrexone at a similar dose has been shown to produce sustained analgesia in mice [21, 22].

### Vaccines and drugs

Mice were immunized with OXY-KLH consisting of oxycodone attached to a tetraglycine linker at the C6 position, conjugated through the linker’s carboxyl terminus to the carrier protein keyhole limpet hemocyanin (KLH) [18]. This immunogen was adsorbed to aluminum hydroxide (Alhydrogel, Invitrogen, San Diego, CA) and administered at a dose of 25 μg. Immunization with OXY-KLH vaccine generates high serum concentrations (300–800 μg/ml) of oxycodone-specific antibodies in mice or rats [17–19, 23]. Native KLH is a large protein of molecular weight 5,000–8,000 kDa consisting primarily of decamers and didecamers of 440 kDa subunits [24]. Vaccine studies involving mice used native KLH as the carrier. To facilitate formulation and analysis of the product, subsequent studies involving rats used immunogen conjugated to GMP KLH subunit dimer (dKLH, Stellar Bioechnologies, Port Hueneme, CA) administered at a dose of 60 ug. The KLH and dKLH conjugates are comparably immunogenic and produce similar serum concentrations of oxycodone-specific antibodies and effects on oxycodone distribution in mice or rats [23]. Control vaccine consisted of carrier protein adsorbed to alum.

Oxycodone HCl (NIDA Drug Supply Program) was administered in 0.9% NaCl. Serum and brain oxycodone concentrations were measured by gas chromatography-mass spectrophotometry as previously described, and all doses and concentrations are expressed as weight of the base. Brain oxycodone concentrations were corrected for brain blood content [18].

General cell culture reagents were obtained from Life Technologies Corporation or Hyclone Laboratories, Inc. (South Logan, UT). Oxycodone-specific murine monoclonal antibody (OXY-specific mAb) was obtained from MyBioSource (San Diego, CA) with a stated purity of 95%.

### Immunologic assays

#### ELISA

Serum oxycodone-specific IgG antibody titers were measured by enzyme linked immunoassays (ELISA) with oxycodone conjugated to BSA (OXY-BSA) as the coating antigen. OXY-BSA was diluted in 0.05 M carbonite buffer pH 9.6, coated onto Costar 9018 EIA/RIA 96 well polystyrene plates (Jackson ImmunoResearch Laboratories, Inc., West Grove, PA), and stored overnight at 4°C. Plates were washed 5 times with 0.05 phosphate buffered saline tween-20 pH 7.2–7.4 (PBST) and repeated between all steps in the ELISA. Plates were blocked with 1% gelatin in PBST for 1 h at room temperature, washed, and stored overnight at 4°C. The next day various dilutions of sera in 0.05 M PBST were added to the wells and plates incubated for 2 h at room temperature. The secondary (detecting) antibodies, Fc-specific goat anti-rat or anti-mouse IgG coupled to horseradish peroxidase (Jackson ImmunoResearch Laboratories, Inc., West Grove, PA), were diluted in 0.05 M PBST, added to the wells, and incubated at overnight at 4°C. The following day enzyme substrate *o*-phenylenediamine (OPD) was used (SIGMA*FAST*^TM^ tablet set, Sigma Life Sciences, St Louis, MO). After 30 min of incubation in the wells, 2% oxalic acid was added to stop the enzymatic reaction. Plates were read at 492 nm on a BioTek PowerWave XS (BioTek Instruments Inc., Winooski, VT). Titers were estimated as the dilution producing 50% maximal binding [18].

#### Competition ELISA

The relative affinities of various drugs for serum oxycodone-specific antibodies were measured by competition ELISA, which differs from the above ELISA assay in two ways. 1) Serum titers measured using ELISA described above was diluted to a fixed 70% maximal binding in the absence of competitor and 2) mixed with competitors that ranged in concentrations from 10^−2^ M to 10^−11^ M diluted in 0.9% NaCl. Relative affinity was determined as the concentration of competitor that reduced the binding of serum antibodies to the plate by 50% and were expressed as the IC_50_ concentration for oxycodone divided by the IC_50_ concentration for the competitor x 100.

### hMOR calcium mobilization assay

The ability of an oxycodone monoclonal antibody (OXY-mAb) to selectively inhibit μ opioid receptor activation by oxycodone was assessed using CHO cells overexpressing both G_α16_, a promiscuous G protein, and the human μ opioid receptor. An OXY-specific mAb was used rather than antiserum generated by the OXY-dKLH vaccine because serum interfered with the assay, as did protein G-purified IgG. The day before assay, wells were seeded (30,000 cells/well) in 96-well, black clear-bottom tissue culture-treated plates (Corning Life Sciences, Tewksbury, MA) and incubated (37°C; 5% CO_2_) overnight. On the day of assay, the cells were washed once with assay buffer (1X HBSS; 0.02M HEPES; 0.781 mg/mL probenecid; 37°C; pH 7.4; Life Technologies Corporation, Grand Island, NY) and incubated for 1 hr at 37°C with 200 μl of half the recommended concentration of Calcium 5 dye (Molecular Devices, Sunnydale, CA). Either the μ opioid agonist oxycodone (10 mM; 100% DMSO) or morphine (10 mM; 100% DMSO) were incubated (30 min; RT) ± Ab (4.38 mg/ml; PBS; MyBioSource, San Diego, CA) at 5x their respective final assay concentrations of 1 μM (EC_50_), 50 nM (EC_50_), and 1:20 dilution. Dubelccos PBS was used for all dilution steps and the final assay DMSO concentration of 1%. At assay, the 96-well plate was placed in a Flexstation^II^ (Molecular Devices) and 50 μl of oxycodone-specific murine monocolonal antibody (OXY-mAb) in duplicate was added to each well at a final concentration of 3 μM and fluorescence intensity recorded (26 read/sec; 60 sec). Peak relative fluorescence units were used to determine the maximum response. Data were graphed using Prism (v7; GraphPad, San Diego, CA).

### Antinociception and respiratory depression

Groups of 16 rats were immunized with OXY-dKLH or control dKLH vaccine containing 60 μg immunogen and 90 μg aluminum as Alhydrogel. Vaccine was administered intramuscularly every 3 weeks for a total of 4 doses. One week after the final vaccine dose baseline antinociception (prior to oxycodone administration) was assessed by placing rats on a 54°C hotplate and measuring the latency to respond, defined as the time until a response of hindpaw lick or jumping [18]. Trials were terminated at 60 sec if rats had not responded. Immediately following the hotplate trial a MouseOx (STARR Life Sciences Corp., Oakmont, PA) arterial oxygen saturation (SaO_2_) monitor was placed via neck collar and baseline SaO_2_ was measured. Rats then received oxycodone every 17 minutes s.c. so that their cumulative oxycodone dose at successive intervals was 1.1, 2.2, 4.5 and 9.0 mg/kg. This oxycodone dose range produced a wide range of antinociception, and the 9.0 mg/kg dose was the highest that could be administered without producing excessive respiratory depression. Fifteen minutes after each oxycodone dose antinociception and SaO_2_ was again measured; this took 2 minutes, resulting in the 17 minutes oxycodone dosing interval. Heart rate was obtained from the oximeter.

### Naloxone reversal of opioid effects

In the antinociception and respiratory depression experiment described above, rats received 0.1 mg/kg naloxone s.c. immediately after the final antinociception and SaO_2_ measures were obtained. Antinociception and cutaneous SaO_2_ were again measured 15 min after naloxone administration. The naloxone dose was chosen based on pilot data indicating that a s.c. dose of 0.01 mg/kg naloxone was not sufficient to reliably reverse oxycodone toxicity in non-vaccinated rats receiving oxycodone 9.0 mg/kg. For perspective, the 0.01 mg/kg naloxone is comparable to the initial naloxone dose used to reverse opioid overdose in humans, and 0.1 mg/kg is comparable to the maximum recommended dose [25].

To determine the rapidity with which naloxone reversed oxycodone respiratory depression, one week after the above experiment the same rats received a single oxycodone dose of 9.0 mg/kg. Thirty minutes later each rat received 0.1 mg/kg naloxone s.c. Arterial SaO_2_ was measured 1 minute before and for 3 minutes after naloxone administration.

### Vaccine immunogenicity with concurrent morphine infusion

Two groups of 12 mice were anesthetized and Alzet® s.c. pumps (model 2006) were placed for morphine or saline infusion. Mice were used because the immunosuppressive effects of morphine are more thoroughly documented for mice than for rats. Morphine or an equivalent volume of saline was delivered at a rate of 8 mg/kg daily, as calculated from the morphine solution remaining in the pump when removed. One week after placement of the s.c. pump both groups of mice were vaccinated s.c. with OXY-KLH 25 μg and 250 μg aluminum as Alhydrogel. A total of 3 vaccine doses were administered at 2 week intervals. Serum was obtained one week after each vaccine dose for measurement of oxycodone-specific antibody titers.

### Vaccine immunogenicity with concurrent naltrexone dosing

Two groups of 7 BALB/c mice received extended-release naltrexone (Vivitrol ®) 50 mg/kg i.m. or saline once on day 0. Both groups were then vaccinated with s.c. OXY-KLH 25 ug/kg in alum every 2 weeks for a total of 3 vaccine doses. Oxycodone-specific antibody titers were measured 1 week after the final vaccine dose and over the following 60 days.

### Data analysis

Latency to respond in the hotplate nociception test, SaO_2_ and serum antibody titers were compared between groups over time by 2-way ANOVA using Sidak’s multiple comparisons test. The effect of naloxone on latency to respond or SaO_2_ within a single group used a two sided paired t test with values before and after naloxone. For the naltrexone/OXY-KLH vaccine experiment, multiple t tests were used rather than ANOVA because of 3 missing data points. All statistics were performed using Prism (v7; GraphPad, San Diego, CA).

## Results

### Vaccine effects on oxycodone-induced antinociception, respiratory depression, and heart rate

**Fig 1:**
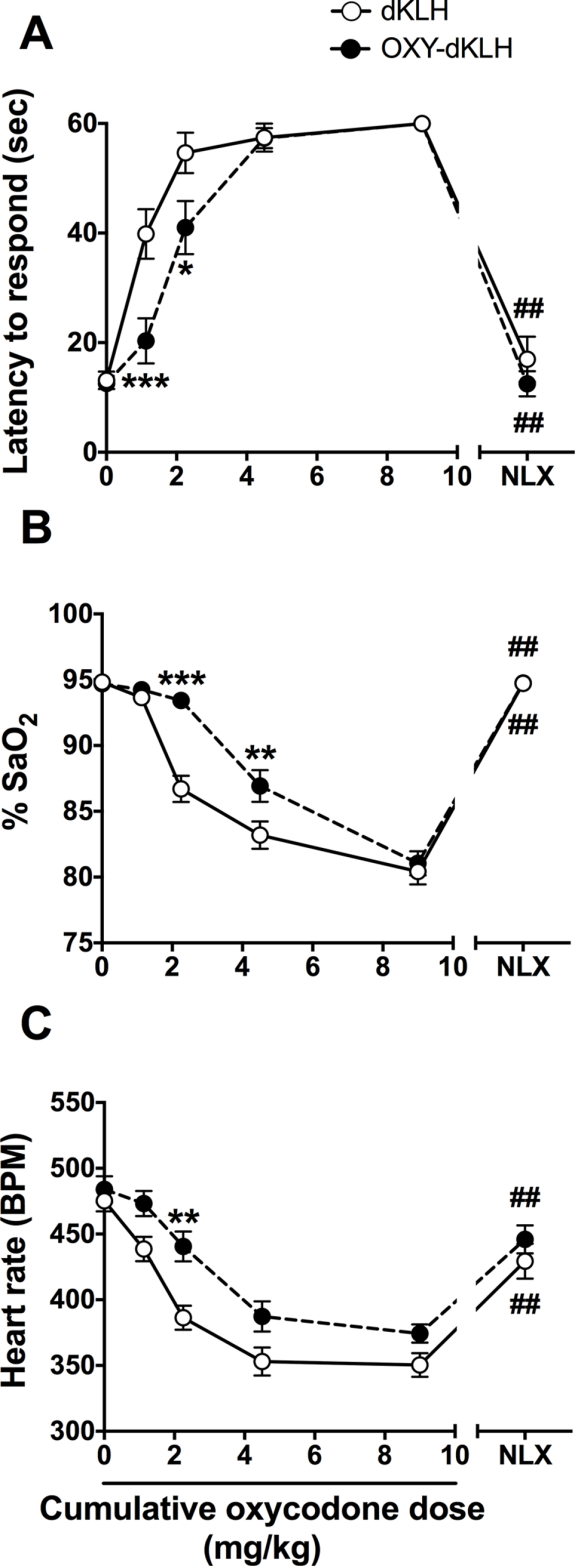
Vaccination inhibits oxycodone-induced antinociception, respiratory depression, and bradycardia. Oxycodone was administered s.c. every 15 min at increasing doses and the doses listed are the cumulative dose received. **A)** Vaccine effects on oxycodone-induced antinociception. Latency to respond is capped at 60 seconds. Naloxone was administered 15 min after the final oxycodone dose. **B)** Vaccine effects on oxycodone-induced respiratory depression measured as SaO_2_. **C)** Vaccine effects on oxycodone-induced decreases in heart rate. * p < 0.05, ** p < 0.01, *** p < 0.001 for the difference between OXY-dKLH and control groups. ## p < 0.01 for the difference (within a single group) between values measured after the 9.0 mg/kg oxycodone dose and after naloxone. There were no differences between groups in latency to respond, SaO_2_, or heart rate following naloxone treatment. Mean ± SEM; n = 15 for KLH and n = 16 for OXY-KLH.

Serum oxycodone-specific antibody titers on days 14, 28, 49, 70 (2 weeks after the first vaccine dose and 1 week after the subsequent vaccine doses) were 54 ± 12, 90 ± 34, 156 ± 64, and 148 ± 85 x 10^3^ respectively (mean±SD). Hotplate antinociception as measured by latency to respond was comparable between groups prior to opioid administration (Fig 1A). The dose-response curve for latency after increasing cumulative doses of oxycodone was shifted to the right by vaccination with OXY-dKLH indicating attenuation of oxycodone-induced antinociception (vaccination, F(1,29) = 7.40, p < 0.05; interaction, F(5,145) = 4.41, p < 0.001; oxycodone dose, F(5,145) = 112.6, p < 0.001). Groups differed in mean latency after the 1.1 and 2.2 mg/kg cumulative oxycodone doses. They did not differ after the higher doses but latency is capped at 60 sec by removal of animals from the hotplate, so that differences might not be demonstrable.

Respiratory adequacy as measured by SaO_2_ was comparable between groups prior to opioid administration (Fig 1B). As with antinociception, the dose-response curve for SaO_2_ was shifted to the right by OXY-dKLH indicating attenuation of respiratory depression (vaccination, F(1,29) = 14.97, p < 0.001; interaction, F(5,145) = 8.13, p < 0.001; oxycodone dose, F(5,145) = 147.5, p < 0.001). Groups differed in mean SaO_2_ after the 2.2 and 4.5 mg/kg oxycodone doses but not at the highest 9.0 mg/kg dose.

Heart rate was comparable between groups prior to opioid administration (Fig 1C). Heart rate was significantly higher in the OXY-dKLH group after the 2.25 mg/kg oxycodone dose, and numerically but not significantly higher at all other times (vaccination, F(1,29) = 11.06, p <0.01; interaction, F(5,145) = 1.66, p = 0.15; oxycodone dose, F(5,145) = 56.59, p < 0.001).

### Naloxone reversal of oxycodone effects

Hotplate antinociception and respiratory depression were completely reversed in both the OXY-dKLH and control vaccine groups by administration of naloxone (Fig 1A and 1B). Both groups showed a significant change toward baseline in these measures after naloxone, and there was no significant difference between groups after naloxone administration. Heart rate was restored to near baseline in both OXY-dKLH and control vaccine groups after naloxone administration (Fig 1C).

**Fig 2:**
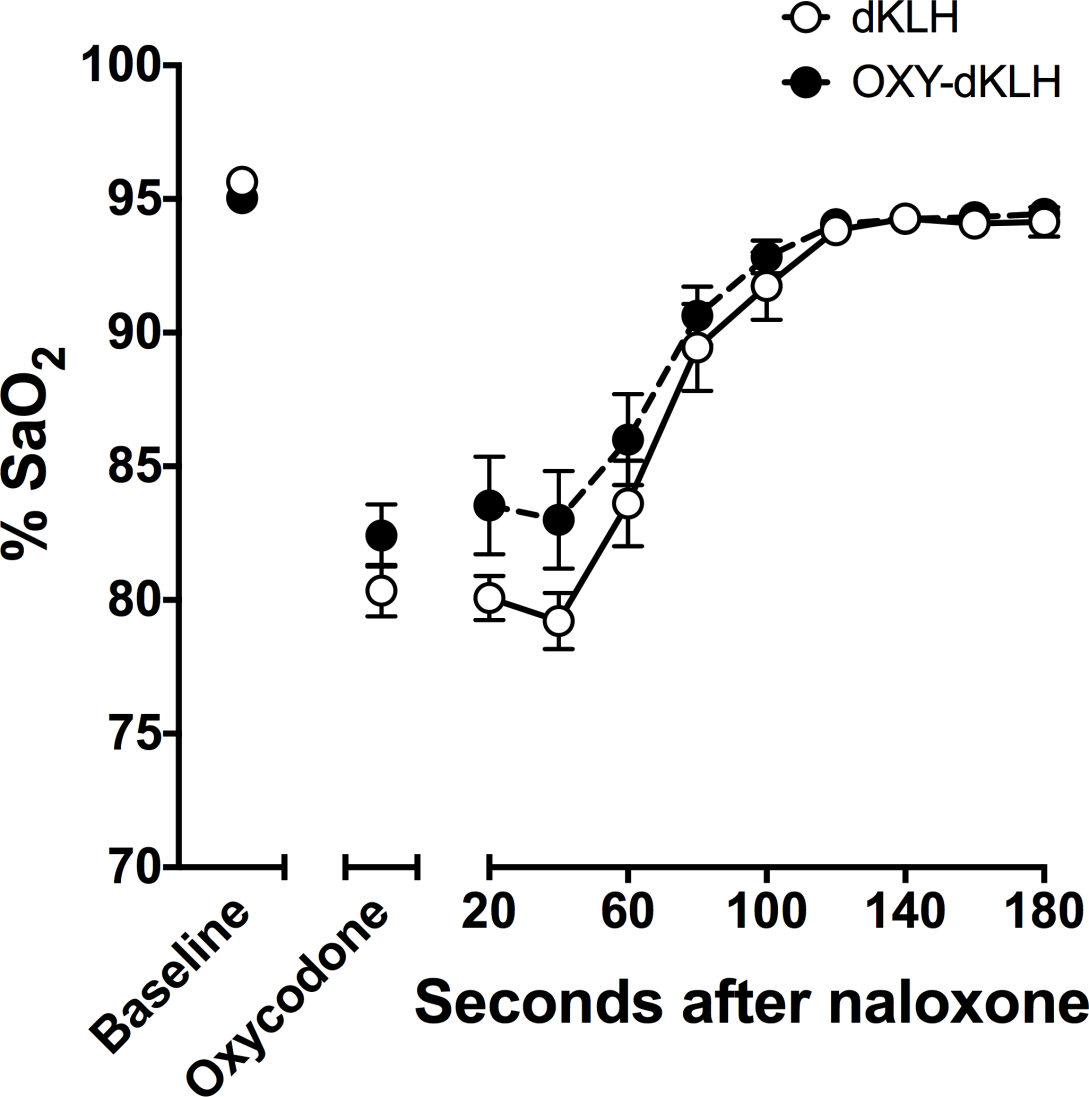
Naloxone reverses oxycodone-induced respiratory depression in vaccinated animals. Naloxone 0.1 mg/kg s.c. was administered to mice 30 minutes after oxycodone 9.0 mg/kg s.c. SaO_2_ recovered promptly in both OXY-dKLH and control groups. SaO_2_ did not differ between OXY-dKLH and control groups at any time after naloxone treatment (p = 0.65). Mean ± SEM; n = 11 for KLH and n = 12 for OXY-KLH.

The rate at which naloxone reversed oxycodone-induced respiratory depression was studied one week later. Rats received a single oxycodone dose of 9.0 mg/kg followed in 30 min by 0.1 mg/kg naloxone. Recovery of SaO_2_ did not differ between OXY-dKLH and control groups, with essentially complete return to baseline and comparable SaO_2_ values within 3 minutes of receiving naloxone (Fig 2).

### Vaccine immunogenicity during concurrent morphine infusion

**Fig 3:**
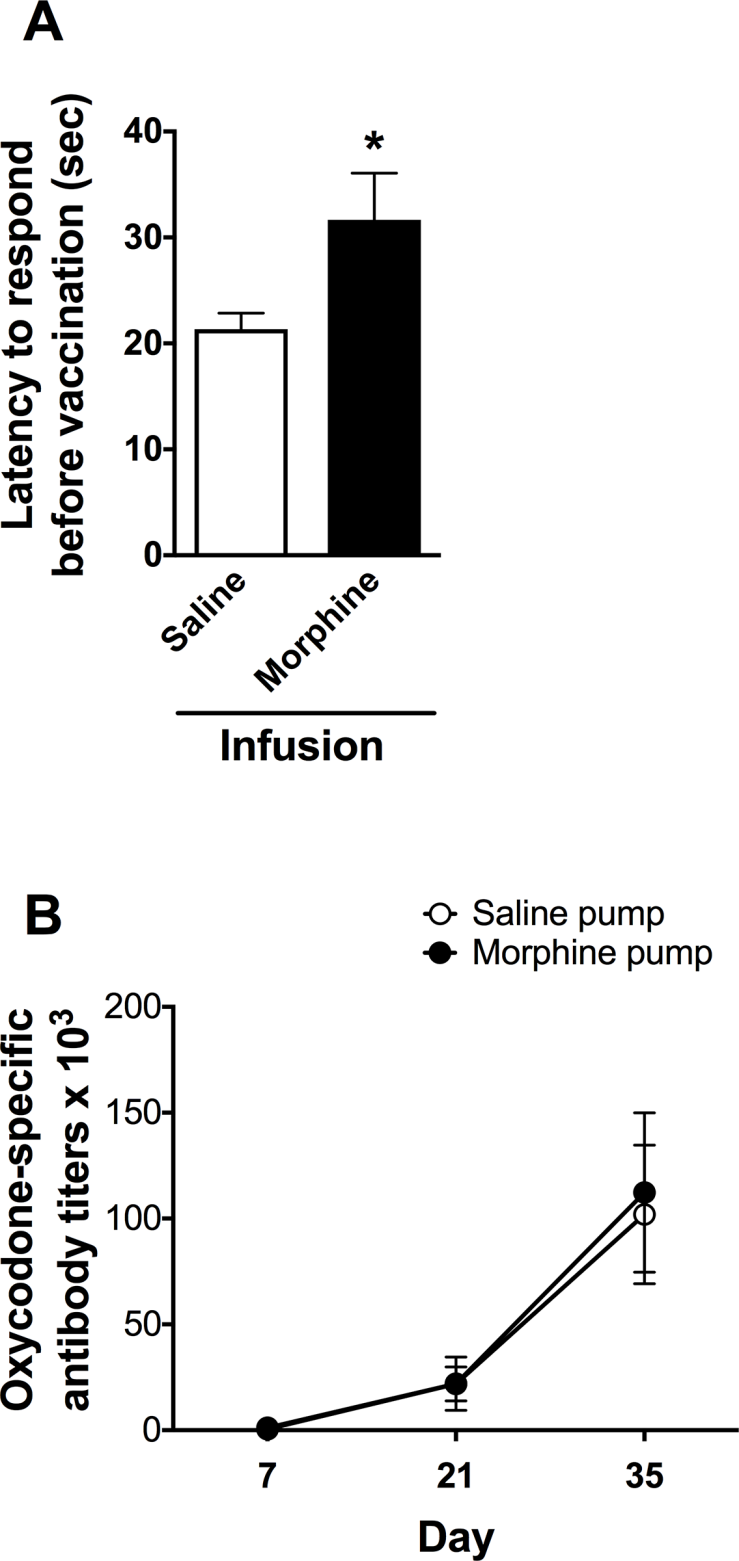
Concurrent morphine administration does not interfere with oxycodone vaccine immunogenicity. **A)** Prior to vaccination, mice receiving s.c. morphine infusion (8 mg/kg/day) showed a significant increase in latency to respond in the hotplate antinociception test (mean ± SEM, *p <0.05). This confirms that mice received a behaviorally active dose of morphine for the following experiment: **B)** After initiation of vaccination, oxycodone-specific antibody titers in serum did not differ between mice receiving morphine infusion (8 mg/kg/day) and those receiving saline (mean ± SD, p = 0.51). For both figures, n = 13 for saline-treated group and n = 12 for morphine-treated group.

To assure that the dose of morphine that mice received in this protocol was clinically relevant, antinociception was tested 1 week after s.c. morphine pump placement (prior to vaccination). The morphine pump group showed a significant increase in latency to respond (t = 2.215, p < 0.05, Fig 3A). Vaccination was initiated after hotplate testing, with vaccine administered on days 0, 14 and 28. Oxycodone-specific IgG antibody titers measured 1 week after each vaccine dose did not differ between OXY-KLH and control KLH groups (t = 0.72, p = 0.48, Fig 3B).

### Vaccination during concurrent naltrexone administration

**Fig 4:**
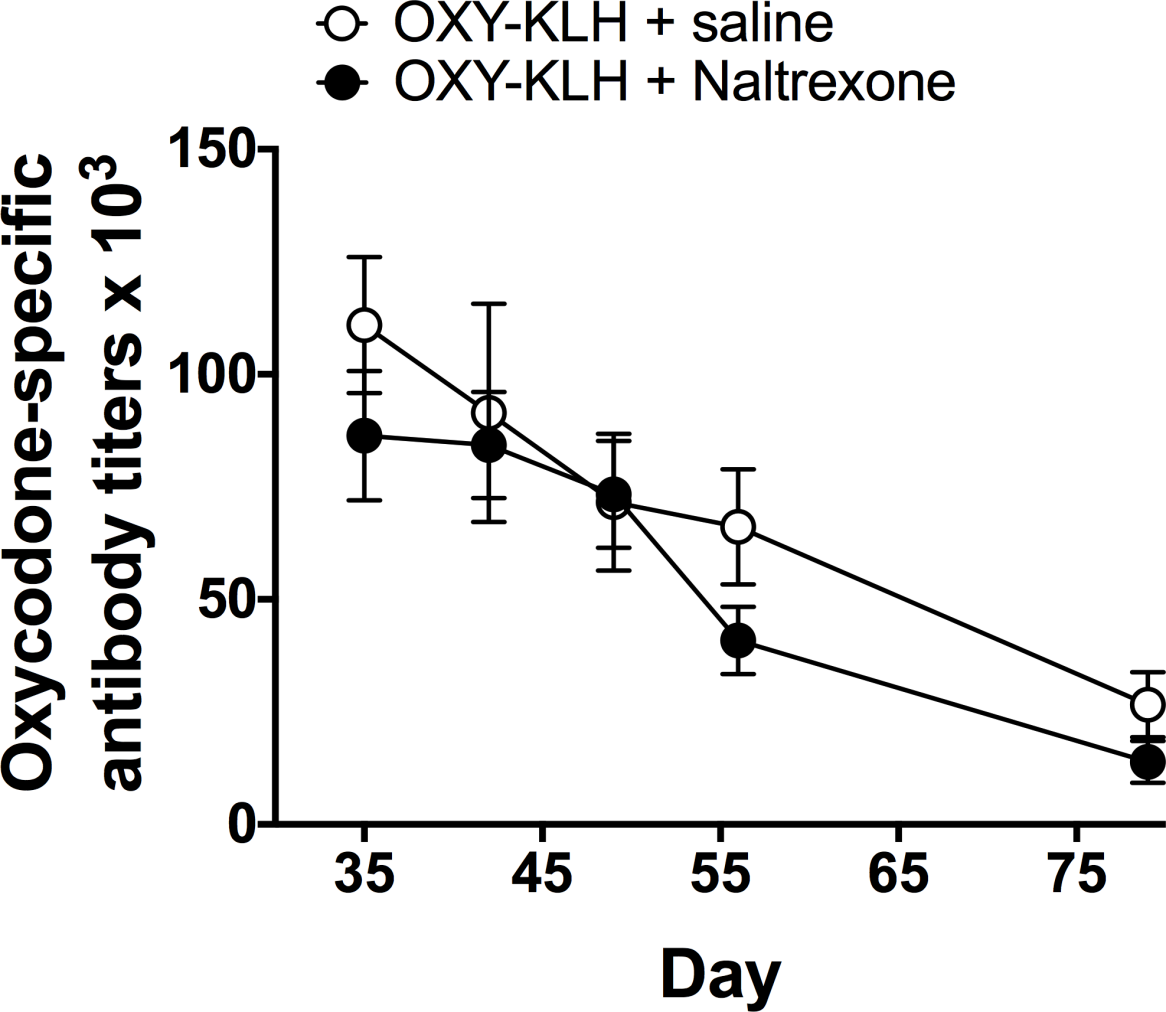
Concurrent administration of extended-release naltrexone does not interfere with oxycodone vaccine immunogenicity. Mice received 50 mg/kg naltrexone i.m. on day -1 and then vaccinated with OXY-KLH on days 0, 14 and 28. Serum oxycodone-specific antibody titers did not differ between groups at any time after completion of vaccination (p = 0.21). Mean ± SEM, n = 4–7/group.

The administration of extended-release naltrexone to mice did not alter their response to vaccination. Oxycodone-specific titers in serum did not differ between naltrexone and control groups at any time after completion of vaccination (p at all time points > 0.1, Fig 4).

### hMOR-mediated calcium mobilization assay

**Fig 5:**
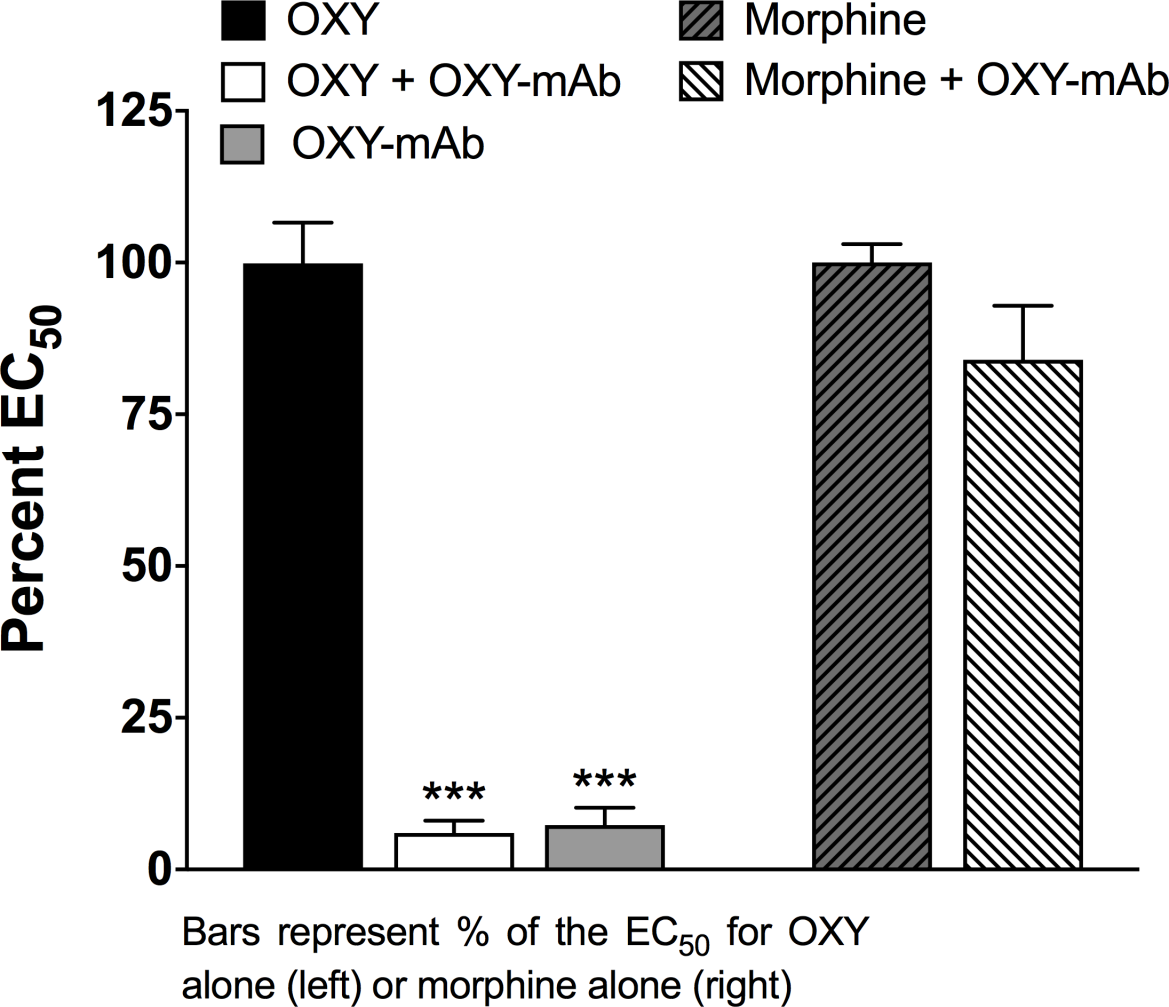
Oxycodone-specific monoclonal antibody prevents oxycodone-induced activation of opioid receptors. OXY-specific mAb blocked OXY-stimulated calcium mobilization but did not block morphine-stimulated calcium mobilization. ***P < 0.001 compared to OXY. Bars represent the % EC_50_ compared to oxycodone alone (left) or morphine alone (right). Mean ± SEM, n = 3/group.

At an OXY-mAb:oxycodone molar ratio of 3:1, OXY-specific mAb abolished oxycodone-stimulated calcium mobilization (Fig 5). Morphine-stimulated calcium mobilization of MOR was not inhibited by OXY-specific mAb, showing the antibody’s specificity for binding oxycodone.

### OXY-dKLH specificity

The relative affinities of antibodies elicited by vaccination of rats with OXY-dKLH, measured by competition ELISA, showed high specificity (high cross-reactivity) for oxycodone and its active metabolite oxymorphone, and low or no measurable cross-reactivity for endogenous opioids, naloxone or naltrexone (Table 1).

**Table 1:** Relative affinities of antibodies generated by vaccination of rats with OXY-dKLH as measured by IC_50_ values in competitive ELISA.

Competitor	IC_50_ (μM)	Cross-reactivity
Oxycodone	0.67	100
Oxymorphone	0.77	87
Naloxone	65	1
Naltrexone	79.2	0.8
Methadone	1900	–
Buprenorphine	1760	–
B-endorphin	–	–
Met-enkephalin	–	–
Leu-enkephalin	8990	–
Dynorphin 1–13	1860	–

To estimate relative affinities (cross-reactivity), IC_50_ values were normalized to that of oxycodone, which is taken to be 100%. Omitted IC_50_ concentrations are >10,000 μM and omitted IC_50_% values are <0.1.

## Discussion

Several medication treatments for opioid addiction are available and in clinical use. Methadone, buprenorphine and naltrexone have each shown efficacy in reducing illicit opioid use and its consequences [26]. Despite their efficacy, each has potential drawbacks, side effects, legal constraints to prescribing, or social stigma associated with their use [27–29]. The result is that many opioid addicts or abusers who might benefit from these medications find them unappealing or unsatisfactory and are not using them [30, 31]. Additional alternative medications that avoid some of these limitations could be helpful in engaging more opioid addicts in treatment.

Opioid vaccines have been developed but none have been administered to humans, with the exception of a heroin vaccine studied in Iran but only sparingly described [32, 33]. However vaccines for nicotine and cocaine have advanced to late stage clinical trails [5]. Their efficacy has to date been limited, most likely due to the moderate and variable titers of antibodies achieved, but their safety has been repeatedly confirmed [34–36]. Adverse effects have been minor and limited to reactogenicity, i.e. short-term effects immediately following vaccination such as soreness at the injection site or mild and short-lived systemic symptoms (aches, malaise) comparable to those observed after other widely used vaccines. This experience confirms abundant animal data and argues for the general safety of vaccination as a strategy for treating drug addiction.

The particular aspects of oxycodone vaccine safety and efficacy analyzed in the current study were chosen based on differences in the actions of opioids compared to nicotine or cocaine, or the clinical setting in which these drugs are used. Each of these challenges must be addressed and understood in order to determine whether opioid vaccines should be considered for clinical trials.

In the current study and others evaluating potential opioid vaccines, hotplate nociception was used as a surrogate for drug reward because it is mediated in the central nervous system and provides a relevant behavioral model of the vaccine’s ability to reduce drug access to brain and its subsequent effects [37]. OXY-dKLH has been shown previously to attenuate oxycodone-induced hotplate antinociception [23]. In the current study, OXY-dKLH also clearly attenuated oxycodone-induced respiratory depression, the predominant cause of death from opioid overdose. Attenuation of respiratory depression was not evident at the highest oxycodone dose, as could be anticipated since it is possible to surmount the effects of this or any other addiction vaccine if the drug dose is sufficiently large. However, the 9.0 mg/kg cumulative oxycodone dose administered is quite large compared to typically abused human intravenous doses, reported to range from approximately 0.15 to 1.1 mg/kg [38, 39]. The protective effect of vaccination at the lower, but still appreciable cumulative oxycodone doses argues that vaccination with OXY-dKLH, while not insurmountable, should provide a substantial measure of protection against oxycodone overdose. These results are supported by a study using an oxycodone vaccine with an alternative design that placed the linker at the bridge nitrogen and used tetanus toxoid as a carrier[40]. This vaccine also produced a rightward shift in the hotplate analgesia assay dose-response curve. This shift was larger than in the current study but it was obtained using mice vaccinated via the intraperitoneal i.p. route, which would not be used in humans. When vaccination was administered s.c. the observed reduction in analgesia was comparable to that reported here. A reduction in mortality after very high doses of oxycodone was reported in mice. These data lend general support to the current findings and the ability of vaccination to reduce oxycodone toxicity. Reduced mortality from fentanyl in mice immunized with a fentanyl vaccine has also been reported [41].

The finding that OXY-dKLH reduces key measures of oxycodone toxicity is also consistent with results of studies with immunotherapies against methamphetamine [42, 43], nicotine [44, 45] and cocaine [46]. Blockade or reduction of seizures, hypertension, hyperactivity, stereotypic behavior and mortality have all been reported. Although some of these studies used passive immunization rather than vaccination, which can produce higher antibody levels than vaccines, these data taken together provide support for the ability of immunotherapies to also provide protection against clinically important toxic drug effects.

A potential concern with this therapeutic approach is that someone vaccinated with OXY-dKLH might deliberately use more than their usual dose of oxycodone to overcome blockade of its effects, but then suffer respiratory depression if the vaccine protects less well against respiratory depression than against the euphoria. Control rats reached 60 seconds latency in the hotplate assay at an oxycodone dose of 2.25 mg/kg while OXY-dKLH rats required 4.5 mg/kg. At these equianalgesic doses the two groups had essentially identical SaO_2_ values of about 87%. The same observation holds true for equianalgesic doses associated with 40 seconds latency; the extent of respiratory depression was the same between groups. These data show that, compared to controls, vaccination with OXY-dKLH reduces oxycodone-induced respiratory depression in proportion to how much it inhibits antinociception, at least over the oxycodone dose range studied. If antinociception is taken as a surrogate for reward, the data suggest that a vaccinated subject using more than their usual dose of oxycodone in order to obtain euphoric effects would suffer no more respiratory depression than they would for the equivalent euphoric effect (from a smaller oxycodone dose) before they were vaccinated. Effects of OXY-dKLH on heart rate were less prominent but also generally protective and support the conclusion that OXY-dKLH attenuates the most dangerous physiological effects of high doses of oxycodone.

The specificity of antibodies generated by OXY-dKLH for oxycodone has been reported previously [18]. In the previous study, the IC50 value for oxycodone was lower than in the current study, likely due to the use of a different coating antigen in the ELISA assay. Both the current a previous study found similar high cross-reactivity of antiserum with oxymorphone, and very low cross reactivity with methadone, buprenorphine, and opioid antagonists. Here we extend ELISA competition evaluation showing no measurable binding of antibody to endogenous opioids. In addition, naloxone completely reversed oxycodone-induced hotplate antinociception and respiratory depression in vaccinated rats, showing that the efficacy of this antagonist for treating clinical opioid overdose is not impaired by vaccination with OXY-dKLH. Both the extent and the rate of reversal of respiratory depression by naloxone were fully preserved in rats vaccinated with OXY-dKLH and were similar to those of controls. Although no saline control was used in the reversal experiment, the complete reversal of respiratory depression occurring within 1–3 minutes of naloxone administration makes spontaneous recovery extremely unlikely. In view of the substantial and increasing incidence of opioid overdose, and the widespread use of naloxone to treat opioid overdose, the non-interference of OXY-dKLH in this regard is absolutely requisite for considering it as a treatment option.

Additionally, an extended-release (30 day) naltrexone formulation used clinically for the treatment of opioid abuse did not interfere with the immunogenicity of OXY-KLH in mice. This result suggests that subjects switching from naltrexone to vaccine or vice-versa for treatment of opioid abuse could do so without interference or the need to stop one before starting the other. It is known that subjects discontinuing naltrexone face a period of high risk for opioid use and also overdose [10, 47]. It is possible that an opioid vaccine could find application in offering some degree of protection during this high-risk period.

The potential for opioids to suppress the immunogenicity of infectious disease vaccines has received some study in humans although most data are observational, and it has been difficult to determine whether the mildly impaired immunogenicity reported in some, but not all, groups of opioid users is due to their drug use or their general health or life style [12, 13, 48]. In the current study morphine was administered to mice by chronic s.c. infusion at a dose within the range of heroin doses that humans might abuse [49] and which has been previously reported to suppress some aspects of immunity in mice [11, 20] or their immune response to injury [50]. We used morphine rather than heroin for this experiment because morphine’s stability is better suited to s.c. pump delivery, and because immunosuppression has been better studied and demonstrated in rodents for morphine. Oxycodone was not used because it has not been well studied in this context to determine whether it is immunosuppressive. No suppression of OXY-KLH vaccine immunogenicity by continuous morphine infusion was observed. These data argue against the possibility that chronic or ongoing opioid use would impair oxycodone vaccine immunogenicity.

The question of whether antibody-bound oxycodone can still activate opioid receptors was addressed using an hMOR-mediated calcium mobilization assay. Monoclonal anti-oxycodone antibody was shown to completely block MOR activation by this measure. A commercially available mAb was studied rather than antiserum from immunized rats because serum was found to interfere with the assay. However the commercial mAb binds in ELISA to oxycodone-protein conjugates synthesized from the same hapten used to immunize rats. It is therefore likely that the commercial mAb and antibodies from vaccinated rats bind similar oxycodone epitopes. These data support physiologic data from in vivo studies showing that the very high concentrations of antibody-bound oxycodone present in the serum of vaccinated animals are inactive [13–17].

A limitation of this study is that mice were vaccinated with a native KLH conjugate while rats were vaccinated with a dKLH subunit dimer conjugate. However the close relationship of these proteins, which differ only in their average degree of aggregation, and their essentially indistinguishable immunogenicity and effects in both mice and rats [23], suggests that the results of the current study generalize to both formulations.

Advancement of OXY-dKLH or any other opioid vaccine to clinical trials remains a complex task. It is recognized that an oxycodone vaccine would face additional challenges in clinical use, as it blocks the effects of only oxycodone and its active metabolite, and possibly hydrocodone [19]. Prescription opioids, principally oxycodone, are responsible for approximately half of opioid overdose deaths in the U.S. [51] but some users switch to other opioids such as heroin when prescription opioid availability is limited and may also be exposed to other opioids used as adulterants. We have previously reported that concurrent immunization with oxycodone and heroin vaccines can block the distribution to brain of both classes of opioids or their active metabolites [52]. It is likely that the eventual clinical use of an oxycodone vaccine such as OXY-dKLH would be as part of such a multivalent vaccine that can target a wider range of abused opioids.

The favorable safety and efficacy data demonstrated in this study for OXY-dKLH support its further development as an additional treatment option for oxycodone abuse. Clinical experience with addiction vaccines, and opioid vaccines in particular, is limited. Studies of these vaccines in early phase clinical trials, including clinical laboratory studies to examine their ability to block opioid effects in a controlled setting, are clearly needed to assess the potential of this strategy as an adjunct to the treatment of opioid use disorder. The high prevalence of opioid use disorder [8] makes the OXY-dKLH vaccine an attractive candidate for further development.
